# H1.0 Linker Histone as an Epigenetic Regulator of Cell Proliferation and Differentiation

**DOI:** 10.3390/genes9060310

**Published:** 2018-06-20

**Authors:** Carlo Maria Di Liegro, Gabriella Schiera, Italia Di Liegro

**Affiliations:** 1 Department of Biological Chemical and Pharmaceutical Sciences and Technologies (STEBICEF), University of Palermo (UNIPA), I-90128 Palermo, Italy; carlomaria.diliegro@unipa.it (C.M.D.L.); gabriella.schiera@unipa.it (G.S.); 2 Department of Experimental Biomedicine and Clinical Neurosciences (BIONEC), University of Palermo, I-90127 Palermo, Italy

**Keywords:** linker histones, histone H1.0, RNA-binding proteins, extracellular vesicles

## Abstract

H1 linker histones are a class of DNA-binding proteins involved in the formation of supra-nucleosomal chromatin higher order structures. Eleven non-allelic subtypes of H1 are known in mammals, seven of which are expressed in somatic cells, while four are germ cell-specific. Besides having a general structural role, H1 histones also have additional epigenetic functions related to DNA replication and repair, genome stability, and gene-specific expression regulation. Synthesis of the H1 subtypes is differentially regulated both in development and adult cells, thus suggesting that each protein has a more or less specific function. The somatic variant H1.0 is a linker histone that was recognized since long ago to be involved in cell differentiation. Moreover, it has been recently found to affect generation of epigenetic and functional intra-tumor heterogeneity. Interestingly, H1.0 or post-translational forms of it have been also found in extracellular vesicles (EVs) released from cancer cells in culture, thus suggesting that these cells may escape differentiation at least in part by discarding H1.0 through the EV route. In this review we will discuss the role of H1.0 in development, differentiation, and stem cell maintenance, also in relation with tumorigenesis, and EV production.

## 1. Introduction

Eukaryotic DNA is complexed with positively charged proteins called histones, to form a highly ordered structure known as chromatin. The basic unit of chromatin is the nucleosome, a complex structure in which 147 base pairs of DNA are wrapped around a core octamer, formed by two molecules each of the core histones H2A, H2B, H3, and H4 [1,2,3,4,5]. The array of nucleosomes (also known as beads-on-a-string) can be further condensed into a supranucleosomal structure (chromatosome), thanks to interactions of DNA in between nucleosomes (linker DNA) with a 5^th^ class of basic proteins: H1 linker histones [6,7,8,9,10,11,12]. Further compaction of the fiber into higher order structures generates interphase chromatin, in which both short- and long-range interactions are present, allowing the extremely long and thin DNA molecules to be condensed so that they be accommodated in nuclei. Interestingly, the chromatin fibers are not randomly distributed throughout the cell nucleus: interphase chromatin corresponding to each chromosome occupies indeed discrete interconnected territories [13,14,15,16] and shows a modular organization in “topologically associated domains” (TADs), delimited by sharp boundaries [17]. In general terms, TADs can assume four main chromatin forms: (i) active chromatin (highly accessible, and decondensed: it contains most active genes); (ii) Polycomb-repressed chromatin (forms a compact environment, well separated from active chromatin); (iii) null (or black) chromatin (highly repressed, and enriched in lamin), and (iv) constitutive heterochromatin (that contains 10-fold less genes than the rest of genome; these genes are, however, actively transcribed and often encode non-coding RNAs) [17].

The hierarchical organization of chromatin can be modulated both during development and in adult cells by at least three mechanisms, that also act in combination: (i) covalent post-translational histone modifications (such as acetylation, methylation, phosphorylation, ADP-ribosylation, ubiquitination, etc.) [18,19], (ii) nucleosome remodeling by ATP-dependent complexes [20,21], and (iii) synthesis and incorporation of specific histone subtypes [22,23,24]. These modifications can affect DNA–histone, and histone–histone interactions, as well as both histone and DNA interactions with a number of other enzymatic and structural proteins. The local distribution of histone modifications in chromatin constitutes indeed a sort of code [25], that can be created by modifying enzymes, indicated as “writers”, recognized by proteins indicated as “readers”, and removed, under changing conditions, by enzymes indicated as “erasers” [26]. Interestingly, on the basis of results obtained with Förster resonance energy transfer (FRET) and protein-induced fluorescence enhancement (PIFE), it has been recently proposed that H1 histones (and H1.0 in particular) do not detach from linker DNA upon binding of transcription factors; the nucleosome indeed remains dynamic even in the presence of bound linker histones [27,28].

As expected, given their basal and universal function in all eukaryotic organisms, histones are highly conserved proteins. However, two different classes of histone genes are present in most eukaryotes. The first class of histone proteins are synthesized only during the S phase of the cell cycle (replication-dependent subtypes), from intron-less genes clustered, in the human genome, on chromosome 6 [29]. The second class of histone proteins, or replacement variants, are synthesized at any stage of the cell cycle (replication-independent subtypes) [30,31]. Thus, in spite of interspecific conservation, each class of histone proteins shows intraspecific variation.

Among the different classes of histones, the most divergent are the linker H1 histones [32]. Eleven H1 subtypes are known in mammals, seven of which (H1.0 (H1°); H1.1 (H1a); H1.2 (H1c); H1.3 (H1d); H1.4 (H1e); H1.5 (H1b); H1.X (H1x)) are expressed in somatic cells, while four are germ-cell specific (H1t, H1T2; H1LS1, and H1_00_) [33]. The expression of H1 variants is differently regulated during mammalian development and in differentiated tissues [33,34,35,36], and different variants have been reported to bind to the nucleosomes in distinct orientation [37], with different affinities [38], probably based on a small number of residues in the globular domain [39], and with a consequent difference in the structure of the condensed nucleosome arrays. In particular, on the basis of atomic force microscopy (AFM) results, H1 subtypes have been classified as weak condensers (H1.1 and H1.2), intermediate condensers (H1.3), and strong condensers (H1.0, H1.4, H1.5, and H1x) [40]. Moreover, it has been suggested that the subtypes of H1 are not uniformly distributed across the genome [41], and can differently affect gene regulation [42,43], also acting as specific rather than global regulators of gene expression; in this context, it was also reported that H1.0 repressed more genes than other H1 variants [44].

In this review, we will focus on H1.0 linker histone, that is mainly expressed in differentiated and non-dividing cells. The role of H1.0 will be discussed in the light of the recent discovery that its levels are modified in cancer, and also of the finding that it can be expelled from cancer cells by loading it into extracellular vesicles (EVs).

## 2. H1.0 Linker Histone in Mammals: Structural Peculiarities in Comparison with the Other Linker Histones and its Localization in Chromatin

All metazoan H1 linker histones have a common general structure, that includes a short N-terminal domain (NTD), a central globular domain (GD), and a long, lysine-rich, C-terminal domain (CTD). The most conserved of them is GD, while NTDs and CTDs show higher sequence divergence [9,33]. Both GD and CTD are required for high-affinity binding to DNA, while the NTD seems to have a less fundamental role; however, its deletion can alter binding affinity [33,45,46]. Interestingly, in aqueous solution, the CTD prevalently assumes random coil and turn-like conformations, but it folds cooperatively as soon as it starts interacting with DNA [47,48]. It has been also reported that CTD folding in the presence of neutral detergents generates secondary structures similar to those observed in H1-DNA complexes, thus suggesting an important role of hydrophobic interactions in the folding pathway [49]. Intriguingly, folding of the fully phosphorylated CTD, in the presence of the anionic detergent SDS, gives rise to an all-β protein, able to rapidly form amyloid-like fibers [49]. Possibly in relation with this property, H1 histones have been also found in the cytoplasm and in the membranes of neurons and astrocytes in prion and Alzheimer’s diseases [50], and they seem to interact with the Aβ peptides [51]. This latter interaction has been also confirmed in vitro [52]. On the other hand, in endocrine and neuronal cells, nuclear H1.0 as well as H3 core histone, and lamin interact with a nuclear fraction of the cellular prion protein [53]. From a more general point of view, many authors have found extranuclear [54], and even extracellular H1 (see Section 5) [55].

**Figure 1:**
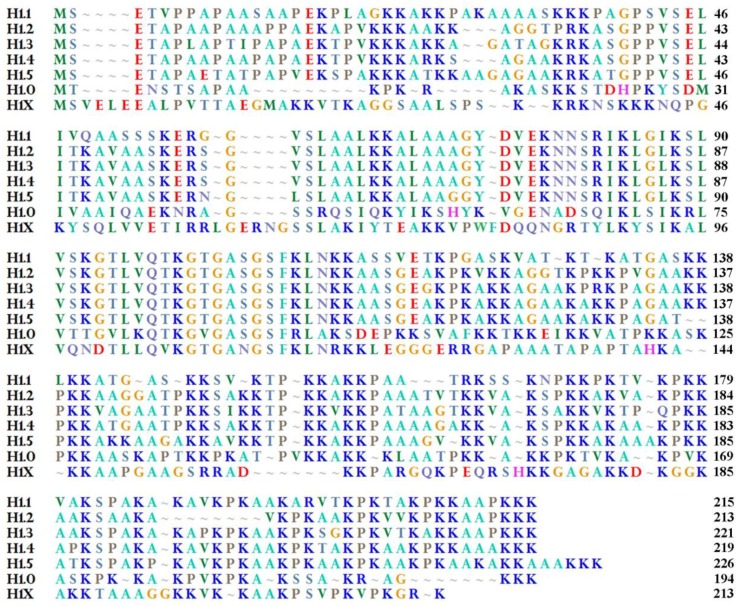
Alignment of all human somatic H1 linker histones. NCBI reference sequences reported: H1.1 (NP_005316.1); H1.2 (NP_005310.1); H1.3 (NP_005311.1); H1.4 (NP_005312.1); H1.5 (NP_005313.1); H1.0 (NP_005309.1); H1.X (NP_006017.1). Alignment of the shown sequences has been done by Bioedit sequence alignment editor [74].

As reported in Figure 1 for the human proteins, all the somatic H1 variants are around 200 amino acid long, with H1.0 being the shortest one [38,40]. The short H1.0 CTD is intrinsically disordered and can interact both with DNA and other proteins [56,57]. Moreover, like the CTD of other H1 variants, it can undergo phosphorylation, a modification that affects its ability to condense chromatin [58]; in particular, three cyclin-dependent kinase (CDK) consensus sequences have been recognized in it, which are reversibly phosphorylated in most cell types [59]. Perhaps phosphorylation at these sites can influence H1.0 ability to bind membranes and/or to exit the nucleus.

**Figure 2:**
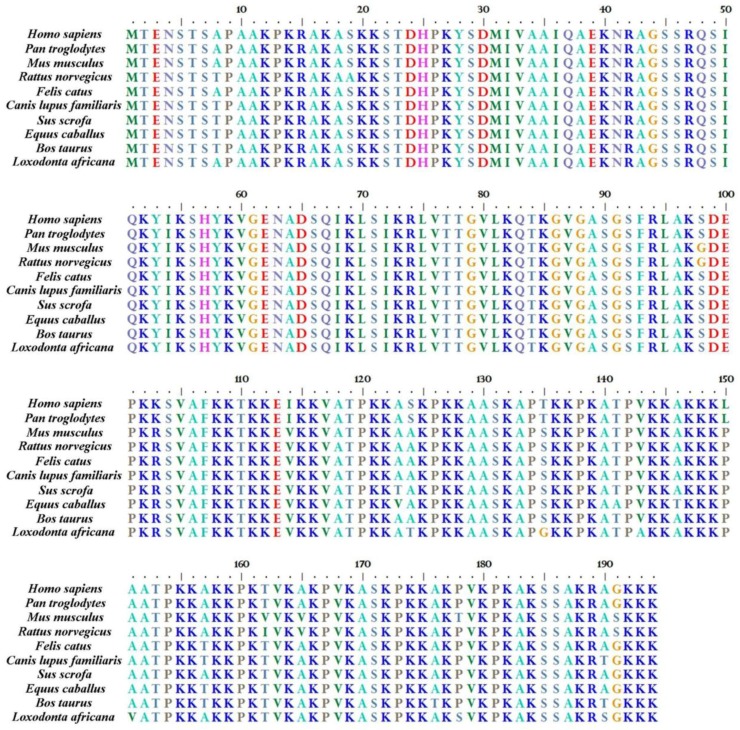
Alignment of H1.0 histones in different mammalian species. NCBI reference sequences reported: *Homo sapiens* (NP_005309.1); *Pan troglodytes* (XP_009436643.1); *Mus musculus* (NP_032223.2); *Rattus norvegicus* (NP_036710.1); *Felis catus* (XP_006934092.1); *Canis lupus fam.* (XP_005625954.1); *Sus scrofa* (XP_003126085.1); *Equus caballus* (XP_005606674.1); *Bos taurus* (NP_001069955.1); *Loxodonta africana* (XP_010597636.1). Alignment of the shown sequences has been done by Bioedit sequence alignment editor [74].

As mentioned in the Introduction, similarity of corresponding variants among species is higher than similarity among different variants in the same species. In particular, H1.0 is the most conserved one (Figure 2). Moreover, these histones, or related proteins, are found in other vertebrates [60,61,62,63].

Interestingly, the gene encoding H1.0 is found on a chromosome (chromosome 22, in the human genome: *H1F0* gene) different from the one (chromosome 6, in the human genome) in which the genes encoding core histones and all the other somatic H1 variants (with the exception of the gene encoding H1X) are found. Moreover, while the mRNAs encoding the other somatic H1 are normally transcribed in replication-dependent way, are not modified by polyadenylation, and are characterized by a stem-loop in the 3’-untranslated region (3’-UTR), H1.0 mRNA is replication-independent and polyadenylated [64,65]. Actually, H1.0 histone is the most abundant variant at nucleoli-associated DNA domains (NADs), rDNA, and other repeated sequences involved in nucleolar organization [41,66]. Recently, an increasing importance has been recognized to nucleoli in very different processes other than the well-known function in ribosome biogenesis; in particular, nucleoli seem to be also involved in processes such as cell cycle control, DNA repair, cell senescence, and apoptosis; thanks to the results of biochemical and proteomic approaches, it has been suggested that the nucleolar H1 histones, and H1.0 in particular, are part of a large protein–protein interaction network which includes core splicing factors, and proteins involved in rRNA biogenesis and in cellular transport [56,67,68]. For example, H1.0 interacts with U2AF35, U2AF65, two SR proteins, and nine heterogeneous nuclear ribonucleoproteins (hnRNPs), thus suggesting that linker histones may regulate mRNA splice site recognition [67].

It has been also found that H1.0 is less concentrated on the rRNA gene promoters and rRNA coding regions than on intergenic regions. By constructing chimeric histones that contain a mosaic of different NTD, GD, and CTD, Okuwaki and colleagues [69] have shown that the GD of H1.0 is required for the enrichment of H1.0 at the intergenic regions of rRNA genes. Interestingly, the GD alone is not sufficient for establishing the binding site preference: at least one of the other two domains is also required; moreover, the preferential binding of H1.0 in the intergenic regions was lost by mutating Lys52 to Glu [69].

As mentioned in the previous section, the binding of transcription factors to linker DNA does not require H1.0 dissociation from DNA. On the other hand, the ability of H1.0 to repress transcription factor (TF) binding can be modulated by acetylation of a specific lysine of the H3 core histone (H3K56), which is located close to the H1.0 binding site [28].

The mRNA encoding H1.0 linker histone shows a long 3’-UTR, containing recognition sites for RNA-binding proteins (RBPs) [70,71], probably involved in the regulation of H1.0 synthesis during development and differentiation. The same region might be also involved in the transfer, mediated by extracellular vesicles (EVs), of proteins able to bind both RNA and DNA; proteins of this kind (see below) might use their ability to bind RNA for accessing EVs and, in turn, cells that surround the EV-producer one; once in the receiving cells, the same proteins might bind DNA, thus modifying its transcriptional potential [72,73].

## 3. H1.0 Expression in Development and Differentiation

During oogenesis, and until the 4-cell stage, somatic H1s are virtually absent from mouse oocytes, except for the H1.0 variant; oocyte nuclei can be indeed stained using an antibody against this histone. Authors’ conclusion is that oocyte would behave as somatic cells, with the other somatic H1s reassembled onto chromatin during cleavage stages [75].

Intriguingly, mice missing H1.0 are fertile and develop normally, suggesting that H1.0 is not required during early embryogenesis, and/or that H1 histones have partially redundant functions [76]: indeed, the specific knockout of each H1 variant in mouse does not cause clear mutant phenotypes, maybe thanks to compensatory mechanisms by up-regulation of other H1 subtypes [77]. In spite of the partially redundant function of linker histones, however, in mouse, the triple deletion of H1c, H1d, and H1e, the major somatic H1 variants, causes a strong reduction of the total amount of H1 and embryonic death at midgestation. On the other hand, triple deletion of H1.0, H1c, and H1e can sometimes allow mice to go through embryogenesis, and, when they survive, they have an apparently normal development and are fertile, even if they grow smaller [78]. Even though single H1 subtypes do not appear necessary for development, different studies have shown that individual histones are involved in the regulation of specific genes in distinct cell types [79,80].

Since the four-cell stage and through the early embryogenesis, when the cells of the mouse embryo divide rapidly and DNA replication is fast, H1.0 level is reduced [75], and the protein is found only in postmitotic lens fiber cells and in nucleated erythrocytes [81]. During embryogenesis, H1.0 increases in a few cell types that undergo differentiation, and, after birth, constitutes 25–30% of total H1 in different tissues [82]. Similarly, in rat embryos, the protein has been shown to appear only in differentiated cells, and in particular in post-mitotic cortical neurons [83,84,85], suggesting that the H1.0 role could be the maintenance of the differentiated state [86]. Mouse embryo extracts from E10.5 contain a small amount of H1.0, with a very low H1.0-to-nucleosome ratio, paralleling the rapid cell proliferation that characterizes this developmental stage [78]. The following increase of H1.0 is accompanied by the increase of H1e, while H1a, H1c, and H1d decrease in the course of tissue maturation [78].

In actively proliferating tissues, such as thymus and spleen, H1.0 is instead kept at low levels [33]. In neonatal mouse liver, H1.0 and H1e represent 9.5% and 19% of total H1, respectively, but their percentages reach 29% and 40% in the adult liver [78]. A constant postnatal H1.0 increase has been described also in rat cerebral cortex [83], and in differentiating dendritic cells [87]. As told before, the deletion of H1.0 generally does not affect differentiation in most tissues, but the function of the dendritic cells in mutant mice is specifically impaired [87]. In mouse differentiating retinal cells, along with H1.0 and H1e, also the expression of H1c increases. Linker histone increase induces a switch in the H1-to-nucleosome ratio up to 1.3, and the nucleosomal repeat lengthens from 190 to 206 bp [88]. In general, the chromatin of newborn rats contains a very small amount of Hl.0, the concentration of which increases during terminal differentiation, for example of neurons, thanks to new synthesis of the protein [89]. At the same time, the concentration of H1.0 messenger decreases from the embryonal day 18 to the postnatal day 10, suggesting that H1.0 expression is regulated also at the post-transcriptional level [90]. In the adult rat brain, H1.0 is not distributed in a homogeneous fashion, and some regions, i.e. cerebral cortex, hippocampus, and thalamus, contain a higher amount of the protein. H1.0 is especially abundant in pyramidal cells of the motor area, while it is much reduced in epithelial cells of the choroid plexus and in Purkinje cells [85].

Interestingly, mice bearing a transgene encoding β-galactosidase controlled by the H1.0 promoter show early expression of the β-galactosidase in brain, retina, and in some blood vessels. This result was confirmed for the endogenous H1.0 gene, suggesting that H1.0 expression is not limited to differentiating cells, or to cells characterized by a low proliferation activity [91]. Moreover, H1.0 may be expressed in the nuclei of cat retinal cells even before their terminal differentiation [92].

The relationship between cell cycle/differentiation and H1.0 synthesis depends on specific elements present in the gene promoter. In the ‘90s, three cis-acting regulatory sequences were recognized to contribute to maximal promoter activity [93,94,95]; two of these elements (the upstream conserved element, UCE, and the H1 box) are highly conserved in all vertebrate replication-dependent H1 genes [95,96]; the third element, called H4 box, is similar to an element (H4 site II) present in the promoters of the genes encoding the core histone H4, where it is involved in the cell cycle-dependent control of H4 synthesis [97]. The H4 box is a unique feature of the differentiation-dependent H1 genes [98]; by using a yeast one-hybrid screen strategy, Lemercier and colleagues [95] identified the high-mobility-group (HMG) box protein (HBP1) as an H4 box-binding factor; moreover, they found that the retinoblastoma protein (Rb) is also involved in the regulation of H1.0 promoter. Therefore, HBP1 and Rb probably mediate expression of H1.0 in relation to the cell cycle, differentiation, and chromatin remodeling.

In addition to regulation of its synthesis during development and maturation of organs and tissues, H1.0 histone also undergoes regulation in adult animals, and particularly in glands that require specific hormones for their maintenance and activity. In 1982, Gjerset and colleagues [81] reported that four days after hypophysectomy, H1.0 was lost in thyroid, adrenal cortex, and testes of rats, although no appreciable general loss of H1 histones or atrophy of the tissues could be noticed. On the other hand, if, after deprivation, the missing hormone, for example thyrotropin (TSH), was injected daily intraperitoneally, H1.0 reappeared [81].

These early observations suggested that the gene encoding H1.0 could contain, in its promoter, sequences responsive to hormones. Cloning and sequencing of the 5'-flanking region of the human gene allowed indeed, in addition to the above-mentioned sequences, characterization of elements consisting of two half-sites arranged as a direct repeat with a short spacer. These motifs were reported to form complexes with different nuclear receptors, thus suggesting that several signal transduction/hormonal pathways can influence H1.0 expression [94,99].

Hormonal dependence of H1.0 gene expression has been also confirmed in developing rat brain by hormone-dependent differences of H1.0 levels in the brain of female and male rats [85].

## 4. H1.0 in Stem Cell Pluripotency Regulation and in Cancer

Pluripotent embryonic stem cells (ESCs) have the potential to differentiate into cells of all germ layers and are consequently of great interest for their potential application in tissue engineering. Given the central importance of the epigenetic architecture of chromatin in determining the transcriptional potential of the cell nucleus, it becomes fundamental to understand the determinants of such an organization in stem cells.

For example, ES cells display specific histone modifications at the level of the so called ‘bivalent domains’, in the promoter of important developmental genes; in particular, these domains are characterized by the simultaneous presence of the H3 histone trimethylated at Lys-27 (H3K27me3: a mark of transcriptional repression) and of H3 di/trimethylated at Lys-4 (H3K4me2/me3: a mark of activation). This combination could mark key developmental genes in ESCs for silencing, giving them, at the same time, the potential to be activated upon induction of a developmental pathway [100,101]. At the same time, a rapid exchange of H1 proteins appears to be required for ESC differentiation. Interestingly, the H1.0 gene promoter contains bivalent domains (H3K4me2 and H3K27me3) in pluripotent cells, suggesting that this variant plays an important role in these cells [102]. Indeed, as discussed below, H1.0 protein has been consistently reported to be involved in the regulation of the “maintain pluripotency-or-differentiate” decision of ESCs, also in the context of cancer growth.

### H1.0 and Pluripotency

It has been known since long ago that H1.0 is predominantly found in tissues with a low level of cell proliferation [81,103,104]. At the same time, it was found that H1.0 expression was also regulated during tissue regeneration [105,106]; in regenerating rat liver, H1.0 decreases to one third after the onset of proliferation [81], and its accumulation does not seem directly dependent on the arrest of cell proliferation, but rather related to a low rate of cell growth [107].

More recently, by using as a model HeLa cells treated with sodium butyrate to induce cell cycle arrest in G0/G1 phase, Happel and colleagues [108] observed, as expected, an increase of the H1.0 mRNA and protein, accompanied by a decrease of mRNAs encoding the replication-dependent variants. Interestingly, in the same study, as well as in others, an uncoupling has been reported between mRNA and protein accumulation, thus confirming the existence of post-transcriptional levels of H1.0 expression regulation [108,109,110].

By using a mouse knock-in system, coupled with chromatin immunoprecipitation and sequencing, Cao and colleagues [111] reported that overexpressed H1.0 displays differential binding at specific repetitive sequences, when compared with H1d and H1c. This finding was confirmed in a genome-wide analysis that showed that overexpressed FLAG-tagged H1.0 was distributed, similarly to H1.2 and H1.3, at the level of the major satellites; it, however, was also enriched at minor satellites and LINE-1 elements [66]. In general, in undifferentiated wild type ESCs, endogenous H1.0 protein is present at very low levels, and the Authors hypothesized that also the genome-wide localization of H1.0 may differ significantly in ESCs induced to differentiate [111].

According to this idea, pluripotent cells have lower levels of H1.0 than differentiated ones, in which H1.0 mRNA represents about the 80% or all mRNAs encoding H1 linker histones [66,102]. A similar difference is clearly seen when we compare the levels of mRNAs encoding, for example, the replication-dependent H1a (H1.1) and H1.0 in mouse ESCs and in a differentiated tissue such as liver; in the liver, H1.0 represents up to the 27% of total H1 [112]. Interestingly, the knockdown of H1.0 in human ESCs does not affect self-renewal but impairs differentiation; moreover, during ESC differentiation in vitro, H1.0 accumulates at specific pluripotency and differentiation genes [102].

Actually, the fact that expression and localization of H1 subtypes are tightly linked to chromatin remodeling suggests their involvement in the chromatin structural transitions that accompany reprogramming [113]. Epigenetic reprogramming accompanies, for example, two critical events of the life cycle of mammals: (i) fertilization, when parental genomes undergo extensive chromatin reorganization, and (ii) in primordial germ cells, during embryonic germ line development [114,115]. These findings suggest an inverse correlation between H1.0 concentration and ability of cells to be reprogrammed.

4.2 H1.0 and Cancer Since the end of the ‘70s, many authors have reported that cancer cell lines treated with differentiation-inducing agents showed an increase of the molecule that has been then indicated as H1.0 [116]. B16 murine melanoma cells treated with sodium butyrate, for example, were found to cease to proliferate rapidly and to start to synthesize melanin; this process was reversible, and if butyrate was removed from the culture medium, the cells started again proliferating rapidly; when starting to differentiate, the cells overexpressed H1.0 mRNA, even if the cells were still proliferating; the level of H1.0 mRNA decreased then very rapidly after butyrate withdrawal [117]. Similar observations were done on mouse erythroleukemia (MEL) cells, where H1.0 and H1c sharply increased during in vitro differentiation [118]; in some studies, the changes in the relative amount of H1.0 during MEL cell differentiation seemed to be primarily a consequence of cell cycle arrest [119]. Interestingly, the expression of H1.0 with phospho-mimetic mutations in the putative cdk recognizing motifs dramatically impaired MEL cell differentiation [120].

As a confirmation of the relationship between H1.0 concentration and cell proliferation, it has been reported that in *c-Ha-ras^Val12^* oncogene-transformed mouse NIH 3T3 fibroblasts, the copy number of the oncogene correlated to the degree of chromatin decondensation, with an increase of the nucleosomal repeat length, and a clear decrease of H1.0 histone concentration in chromatin [121].

In more recent years, it is becoming increasingly clear that epigenetic modifications can play key roles in cancer; given that H1 histones are central players in the overall organization of chromatin, much attention is now focused on these histones, on their mutations (both germline and somatically acquired), as well as on their cancer-related interacting partners in the cell. For example, it has been reported that H1 histones are involved in the regulation of DNA and histone H3 methylation, in mouse ESCs, at the sites encoding the long non-coding H19 RNA, and the Gon-Two Like (gtl2) protein. These activities depend on the ability of the C-terminal domain of at least some H1 species to interact with DNA methyl transferases (DNMT) 1 and 3B, and to recruit them on DNA, as well as on H1 ability to inhibit binding of SET domain-containing lysine methyltransferase 7 (SETD7, or SET 7/9), thus inhibiting methylation of H3K4 [122].

In general, variant-specific patterns can be observed in specific cancers, together with additional intra-tumor variability [123]. In particular, H1.0 is downregulated in a variety of cancers. Moreover, its expression is heterogeneous and in most cases correlates with the tumor grade. For example, in a study aimed at analyzing the relationship between H1.0 distribution in breast cancer and the differentiation/proliferation grade of the cells, it was found that in most of the cells with a moderate or high level of differentiation, including those invading connective and adipose tissues, H1.0 was expressed, while in low differentiated tumors the number of H1.0 expressing cells was considerably lower [124]. Similarly, the expression of H1.0 was significantly reduced in ovarian malignant adenocarcinoma respect to benign adenomas [125]. In a study on patients affected by gliomas of different grades, it was found that grade III-IV gliomas had significantly less H1.0 histone than grade II gliomas. Moreover, in a multivariate regression analysis, H1.0 made a small but significant contribution to survival rates, suggesting that H1.0 can have a prognostic value for glioma patients [126].

Now, besides differences among different types and different grades of cancers, it is clear that an intra-tumor heterogeneity also exists, that includes genetic and epigenetic heterogeneity, as well as phenotypic heterogeneity deriving from the properties of the tumor stem cells as well as from the heterogeneity of the microenvironment [127,128]. In 2016, Torres and coworkers [129] have shown that intra-tumor heterogeneity is linked to differential expression of H1.0 histone. The authors had previously shown that in vitro transformation of epidermal fibroblasts generated cells expressing markers of cancer stem cells (CSCs), such as the Stage-Specific Embryonic Antigen 1 (SSEA1); these cells acquire multipotency, and the ability to proliferate indefinitely; moreover, when injected into mouse, they generated hierarchically organized tumors, where some cells retained the CSC potential, while others (SSA1 negative) gave rise to a progeny with moderate proliferating capacity [130]. Starting from this already established system, the authors then compared gene expression in SSA1 negative and positive cells and discovered that H1.0 contributes to determine which cells in the tumor maintain long-term self-renewal potential. In particular, they found that silencing of H1.0 in CSCs is necessary to maintain self-renewal potency [129]. On the other hand, overexpression of H1.0 can efficiently limit proliferation and induce differentiation. These findings suggest that H1.0 can silence genes specifically involved in proliferation, while activating genes involved in differentiation [129].

Torres and colleagues also performed bisulfite sequencing of the H1.0 gene, comparing SSEA1^+^ and SSEA1^-^ cells. They found a CpG-rich region that is methylated and silenced in SSEA1^+^ cells. Similarly, methylation of the region was evidenced in clinically-derived samples expressing low amounts of H1.0. In general terms, cells lacking H1.0 show upregulation of sets of AT-rich genes involved in oncogenic cell responses and stem cell maintenance [129]. As a whole, these data suggest that H1.0 can play a central role in creating a barrier to stemness and to reprogramming [127,129].

Intriguingly, all these discoveries on the importance of H1.0 in stem cell physiology and in cancer are apparently in contrast with the fact that H1.0 knockout mice are viable.

## 5. H1.0 Protein and mRNA as Cargoes of Extracellular Vesicles

It is now universally accepted that both prokaryotic [131,132] and eukaryotic [133,134,135] cells can secrete proteins and other molecules through extracellular vesicles (EVs) of different size and origin: some (ectosomes, or membrane vesicles) originate from domains of the plasma membrane with a process resembling the virus budding, while other vesicles derive from multi-vesicular bodies (MVB), in the endosomal compartment, and are called exosomes [136]. Importantly, for reasons only partially understood, EVs are produced in higher amount by cancer cells and are involved in several tumorigenesis-associated events, such as: (i) suppression of immune response; (ii) angiogenesis; (iii) stimulation of cancer growth, invasion, and metastasis [137,138,139].

In addition, cancer cells might use EVs like trash boxes to discard unwanted molecules [72,140].

In general, EVs contain a variety of molecules involved in their formation and secretion, in membrane targeting, fusion, and trafficking, as well as in delivering them to target cells. Among the proteins, it has been possible, for example, to find chaperones [141], RAB27A [142], and RAB35 [143], cytoskeletal components and signal transducers, as well as cytoplasmic enzymes [144,145,146]. The ability to package a large variety of molecules and to transfer them across cell boundaries probably had a central adaptive role during higher eukaryote evolution: these exchanges of organized material can play a role, indeed, in levelling responses and activities of cell populations in a given tissue [147]. However, under pathological conditions, the same abilities can turn into a way to spread the pathology [148,149,150,151,152,153,154].

Now, as discussed in Section 4, H1.0 linker histone, traditionally associated with cell terminal differentiation, has recently attracted interest for its involvement in generating epigenetic and functional intra-tumor heterogeneity [129] and because of its down-regulation in cancer cells [155]. In contrast to what could be expected, however, H1.0 is synthesized at least in some cancer cell lines. Interestingly, however, it can be discarded from the cells through EVs [72,140]. Moreover, cells can also discard the mRNA encoding H1.0, in a complex with RNA-binding proteins [72].

### 5.1 Sorting of H1.0 Protein and mRNA to EVs

One of the still not completely understood aspects of EV physiology is the specific sorting of molecules to them. Although at least some components of EVs might be loaded passively, just because they occupy portions of cytoplasm that are enclosed into the vesicles, most observations suggest that active sorting processes do exist. A central role is probably played by lipids and by proteins able to bind them. At the same time, the presence of putative RNA-binding domains (RBDs) has been recently reported in a high number of cellular proteins, many of which are basal metabolic enzymes [156], or proteins involved in cell-to-cell and/or cell-to-environment communication [73,157]. Enzymes acting on lipids and normally present in membranes have been also reported to harbour putative RBDs [158].

As mentioned, we found that H1.0 histone is present in EVs released from some cancer cell lines [72,140]. Now, we discussed above the fact that H1 linker histones can also be found on the surface of different cell types [159,160,161], where they probably interact with membrane lipids [49]. Thus, a first possibility to explain sorting of H1.0 histone to vesicles can be its ability to interact with lipids. On the other hand, in the case of melanoma cells, we observed that H1.0 present in EVs (but not that present in total cell lysates) is probably sumoylated, and sumoylation has been already reported as important for sorting to vesicles, for example, of alpha-synuclein [162], and hnRNPA2B1, as well as of hnRNPA2B1-bound microRNAs [163]. Thus, sumoylation and/or other post-translational modifications could be involved in sorting. Probably, more mechanisms could act in alternative or even together to ensure a high efficient delivery of specific molecules to EVs.

Interestingly, H1.0 mRNA is also present in the same EVs that also transport the H1.0 protein. By using a chromatographic approach, and the in vitro transcribed, biotinylated H1.0 RNA as a bait, we found, among the proteins present in EVs released by melanoma cells and able to bind H1.0 mRNA, proteins normally found in membranes (our unpublished results); among these, for example, were: (i) phosphatidylinositol-4-phosphate 3-kinase C2 domain, alpha subunit (PIK3C2A), a protein involved in several signal transduction pathways, and membrane trafficking processes, and (ii) Golgi Apparatus protein 1 (Glg1), a ubiquitous protein involved in membrane trafficking, able to bind fibroblast growth factor (FGF), and E selectin.

On the basis of all these observations, we suggest the possibility that lipids and membrane-binding proteins allow anchoring of a collection of coding (and possibly also non-coding) RNAs that might, in turn, bind other RNAs and proteins (for example, metabolic enzymes), thus creating complexes of molecules ready to be transported outside the cell via EVs. The presence of metabolic enzymes in the complexes might even help to produce locally the energy necessary to curve/modify the membranes in order to produce vesicles. In this context, it is also to be underlined that RNA can also directly bind more or less ordered lipid bilayers [164,165,166].

### 5.2. H1.0 RNA as Carrier of Proteins 

A further function of RNA-protein complexes sorted to vesicles might be based on a possible role of RNA as a carrier of proteins [72,73]. By using the above-mentioned affinity chromatography, followed by MALDI-TOF mass spectrometry, we found, among the H1.0 mRNA-binding proteins extracted from EVs released by melanoma cells, that the most prevalent was the myelin expression factor 2 (MYEF2). This is a nuclear factor that represses the gene encoding the mouse myelin basic protein [167]. MYEF-2 also contains two putative RNA recognition motifs (RRM), which were already known to bind DNA [168]. Finally, it can form a complex with the Runt-related transcription factor 1 (RUNX1), involved in generating hematopoietic stem cells [169]. On the basis of these previous observations, MYEF2 expression in cancer cells could be not surprising. Moreover, since this protein contains RNA-recognition motifs, its binding to an mRNA might be expected.

Now, the fact that MYEF-2, mostly expressed in undifferentiated cells, binds to the mRNA encoding the differentiation-specific H1.0 histone, probably participating in its elimination from the cells via EVs, can shed some light on the biochemical mechanisms involved in tumorigenesis-linked EV production. In addition, we propose that H1.0 mRNA could in turn function as a MYEF2-carrier: once entered a new cell, MYEF2 could indeed also function as a transcription factor, able to induce an epigenetic change of expression of the receiving cell.

## 6. Conclusions and Perspectives

Ever-growing attention is currently reserved to cancer epigenetics; it is indeed clear that, beside either germline or somatic genetic alterations, modifications of chromatin structure in the absence of changes of the underlining DNA sequence can seriously alter cell behavior, leading to tumorigenesis.

Chromatin modifications can derive from aberrant histone post-translation modifications as well as from an altered pattern of DNA methylation, but also from the presence in chromatin of specific histone variants. Among these latter proteins, histone H1.0 has been attracting interest for more than 40 years, since it was found to increase during differentiation and to be downregulated in proliferating and transformed cells of different kinds. More recently, it has been also shown that intra-tumor heterogeneity is linked to differential expression of H1.0 histone [129]. Moreover, it has been reported that at least some cancer cells produce H1.0 but discard it via extracellular vesicles [72,140]. Interestingly, H1.0-encoding mRNA is also present in the same EVs and seems to function as a carrier for proteins able to bind both RNA and DNA [72,73].

On the basis of these results, restoring H1.0 expression might be a target for strategies aimed at reducing proliferation and expansion of self-renewing cells. On the other hand, circulating EVs that carry H1.0 might have a diagnostic value.

In order to go ahead along this way, however, further analyses are required to shed light on the intriguing, observation that, in spite of the critical properties of H1.0, knock-out mice that do not express H1.0 are viable and do not show special alterations.

Moreover, it is necessary to investigate whether H1.0 elimination from cancer cells through EVs is a generalized phenomenon. Finally, it is also of some interest to ascertain whether H1.0 mRNA also has a generalized function as a protein carrier.
